# Linoleic Fatty Acid from Rwandan Propolis: A Potential Antimicrobial Agent Against *Cutibacterium acnes*

**DOI:** 10.3390/cimb47030162

**Published:** 2025-02-27

**Authors:** Florent Rouvier, Lydia Abou, Emmanuel Wafo, Jean Michel Brunel

**Affiliations:** 1 INSERM, SSA, MCT, Aix Marseille Université, 13385 Marseille, France; 2 La Garniere, Route de Mazaugues, Observatoire Français d’Apidologie (OFA), 83136 Mazaugues, France; 3 C2VN, INSERM 1263, INRAE 1260, Aix Marseille Université, 13385 Marseille, France

**Keywords:** antibacterial activity, *Cutibacterium acnes*, fatty acids, linoleic acid, acne, propolis

## Abstract

Acne is a very common skin condition that causes pimples in 80% of adolescents despite the many effective treatments developed. Various compounds have been employed in the treatment of acne, including erythromycin ointments and antiseptics, yielding mixed results. The rise in erythromycin-resistant *C. acnes* strains has driven the pursuit of new antimicrobial agents, especially those obtained from natural sources. Propolis that was collected in Rwanda was extracted, fractioned, and analyzed for its activity against *C. acnes* growth in accordance with NCLSI guidelines. Our work revealed that linoleic acid has a significant effect on *C. acnes* growth at a low concentration (16 µg/mL). A comparison of the antimicrobial activities of a broad panel of well-known fatty acids revealed a specific mode of action for linoleic acid, characterized by a significant membranotropic effect on *Bacillus cereus* established by measuring extracellular ATP levels as an indicator of membrane permeability. Our data suggest that linoleic acid is effective against *C. acnes* and could be a promising candidate for developing a propolis-based ointment for acne treatment.

## 1. Introduction

Propolis, commonly referred to as “bee glue”, is a resinous material produced by honeybees that they gather from various botanical sources, mix it with their own secretions and beeswax, and use it to repair and protect their hives [1,2]. The exact composition (more than 150 different molecules) varies based on the honeybee species, plant source, and season. This misunderstood apiary product has demonstrated a wide spectrum of properties, including anti-inflammatory, anticancer, antioxidant, and antimicrobial effects [3,4,5,6,7,8,9,10,11,12]. This variability leads to challenges in standardizing its chemical profile, making it difficult to establish a consistent understanding of its active compounds. Additionally, propolis contains a diverse range of bioactive components, including flavonoids, phenolic acids, and terpenoids, often in trace amounts. The interplay of these components and their synergistic effects further complicates the identification and quantification of its key constituents. Furthermore, analytical techniques used to study propolis, such as chromatography and spectrometry, may yield inconsistent results depending on the methods and parameters used, contributing to discrepancies in reported compositions [13]. Furthermore, many scientists investigating the biological properties of propolis have continued to assume that the term “propolis” is as chemically definitive as the botanical name of a medicinal plant. However, its composition varies depending on the surrounding flora, and not all types will exhibit the same efficacy. In our pursuit of potent antimicrobial molecules, we explored the potential of a propolis sample collected in Rwanda, a region renowned for housing one of the world’s last remaining primary forests. Our goal was to assess whether the endemic tree species in this country offer a potential source of novel molecules for advancing antimicrobial research, with a particular focus on treating acne, which affects more than 80% of adolescents during puberty [14,15,16,17].

## 2. Materials and Methods

### 2.1. Reagents

Dichloromethane, methanol, ethyl acetate, and petroleum ether were purchased from VWR. Pure samples of fatty acids C10:0, C11:0, C12:0, C16:0, C17:0, C18:0, C22:0, C23:0, C14:1, C16:1, C18:1, C24:1, C18:2, C18:3, and C22:6 were purchased from Sigma-Merck (Saint-Quentin-Fallavier, France) and used as received.

### 2.2. Apparatus

The use of a Teledyne Combiflash device (Serlabo, Entraigues sur la Sorgue, France) allowed column chromatography to be carried out on Macherey Nagel (Hoerdt, France) silica gel (70–230 mesh). Mass spectroscopy was carried out on a GC-MS QP2010S Shimadzu apparatus and using a column Macherey Nagel (Hoerdt, France) Optima 5 MS Accent 725820.30—30 m × 0.25 µm × 0.25 mm (injector temperature: 250 °C; oven temperature: 40 °C; interface temperature: 250 °C; source temperature: 200 °C; acquisition mode: scan from 50 to 600 *m*/*z*; injection volume: 1 µL; temperature gradient: 40 °C for 0 min, then 5 °C increase up to 80 °C for 1 min, then 5 °C increase up to 100 °C for 0 min, then 9 °C increase up to 200 °C for 30 min; analysis time: 54.11 min.)

### 2.3. Preparation of the Hydroalcoholic Extract of OFAP2

Propolis (90 g of sample OFAP2) was collected in hives located in Rwanda in the region of Musha (Rwamaga district). Propolis OFAP2 (9.0 g) was extracted using water–ethanol (30:70, *v*/*v*) with a Biotage Initiator microwave apparatus set to operate at 100 °C and 2 bars for 10 min. Following centrifugation, the resulting ethanol extract was prepared at a propolis concentration of 10 mg/mL and subsequently used for antimicrobial testing.

### 2.4. Bacterial Strains

*E. coli* ATCC25922, *B. cereus* ATCC11778, and *S. aureus* ATCC25923 were purchased from ATCC (Manassas, VA, USA). *S. epidermidis* CIP81.55 and *C. acnes* DSM 1897 were purchased from the Pasteur Institute (Paris, France) and DSMZ (Braunschweig, Germany), respectively. These strains had previously been stored in 15% glycerol (*v*/*v*) at −80 °C for cryoprotection. A colony from a fresh culture of each strain was incubated overnight in BHI broth at 37 °C under agitation. This suspension was then used to prepare the inoculum by diluting 20 to 100 µL of the culture in 3 mL of fresh medium [18,19].

### 2.5. Determination of Antibacterial Activity

A standard microdilution test was utilized to measure the compounds’ antibacterial activity in accordance with Clinical and Laboratory Standards Institute (CLSI) guidelines. To improve reproducibility, the method was slightly modified by increasing the mixing volumes to 200 µL. All the samples (chemical compounds or propolis extracts) were prepared in ethanol (70%) at a concentration of 10 mg/mL.

For each bacterial strain test, a culture colony was incubated in a 5 mL culture tube of brain heart infusion (BHI) broth at 37 °C overnight under agitation at 100 rpm. For the *C. acnes* strain, an anaerobic atmosphere generator (GENbag anaer, Biomérieux (Marcy l’Etoile, France)^®^) was used. Subsequently, 20 to 100 µL of the overnight culture was added to BHI (3 mL) and the tubes incubated at 37 °C for 3–4 h at 100 rpm.

Each sample was tested was subjected to twofold serial dilutions, starting with ethanol (70%) for the first two dilutions, then by water for subsequent dilutions. This process produced working solutions with concentrations of 5000 to 80 μg/mL, corresponding to ethanol levels of 70% to 2.2%. The final concentrations in the microplate wells ranged from 250 to 4 μg/mL, with ethanol percentages decreasing from 3.5% to 0.1%. The ethanol content in the samples was not detrimental, as we demonstrated that the MICs for doxycycline and erythromycin prepared in either water or ethanol yield similar results (Table S1). All experiments were performed in triplicate.

### 2.6. Minimum Inhibitory Concentration (MIC) Determination

According to a previously reported procedure, the MIC is determined as the minimum concentration of the tested product [20] in the well that does not visually show red coloration, indicating bacterial growth inhibition [21]. All experiments were performed in triplicate.

### 2.7. Real-Time Growth Curves

Freshly prepared stock solutions (10 μL each) at concentrations of 40, 80, 160, 320, and 640 μg/mL of the selected compounds were added to a 96-well plate, along with 190 μL of a bacterial suspension of *B. cereus* ATCC11778 containing 5 × 10^5^ CFU/mL in brain heart infusion (BHI) broth. The real-time bacterial growth was measured (OD590 nm) every 20 min by incubation of the plate performed at 37 °C for 18 h in a Tecan Spark Reader. All experiments were performed in triplicate.

### 2.8. ATP Efflux Measurement

Phosphate-buffered saline (PBS) was used to prepare fresh stock solutions of the different fatty acids, which were then 10-fold diluted in BHI.

The *B. cereus* ATCC11778 suspensions were prepared in BHI and incubated at 37 °C until reaching the exponential growth phase. The compound solution was mixed with 90 parts by volume of bacterial suspension in a 96-well white flat-bottomed cell culture plate and briefly shaken for 5 s in a 37 °C incubator. After a 3-min incubation period, 50 μL of luciferin–luciferase reagent was added and luminescence was measured using the Tecan Spark Reader (Tecan, Männedorf, Switzerland) for six readings at 30-s intervals. ATP efflux was quantified at its peak in three independent experiments, using a positive control (squalamine (100 µg/mL)) and BHI as a negative one. All experiments were performed in triplicate.

### 2.9. Statistical Analysis

Based on the results obtained, the Shapiro–Wilk normality test was performed to determine whether the data followed a normal distribution. Subsequently, an analysis of variance (ANOVA) was performed to determine if the means of the different groups were significantly different. When significant differences were found, a Tukey post hoc test was applied to perform pairwise comparisons between the groups and identify which pairs exhibited statistically significant differences (*** *p* ≤ 0.001).

## 3. Results

A sample of propolis OFAP2 (9.0 g) was extracted with ethanol (70%) resulting in an ethanol extract that demonstrated significant antibacterial activity against *B. cereus* ATCC11778 and *C. acnes*, whereas MICs up to 256 µg/mL were encountered against both Gram-positive (*S. aureus* ATCC25923) and Gram-negative (*E. coli* ATCC25922, *P. aeruginosa* PA01) strains (Table 1).

Encouraged by these promising results with *C. acnes*, we aimed to further investigate and identify the molecule or molecules responsible for the observed antibacterial activity. To this end, the crude ethanol extract was concentrated under vacuum into a crude powder (1.6 g) and then subjected to flash chromatography using a gradient from nonpolar to polar solvents, specifically petroleum ether (solvent A) and ethyl acetate (solvent B). This process resulted in 26 fractions of 20 mL each, which were subsequently grouped and concentrated into three fractions, labeled F1 to F3, weighing 195, 550, and 560 mg, respectively.

Subsequent testing clearly showed that only fraction F2 exhibited a notable MIC against both *B. cereus* and *C. acnes* strains, indicating that the active molecules responsible for the antibacterial activity were exclusively present in this fraction of the propolis extract.

Thus, fraction F2 underwent GC-MS chemical profiling (Figure S1), which revealed the presence of numerous compounds in varying proportions, as shown in Table 2. All the compounds were unambiguously identified by comparison with a mass fragmentation pattern library (NIST) and using the NIST 23 GC Method/Retention Index Database, which provides retention indices and gas chromatographic conditions for more than 180,000 compounds.

**Figure 1:**
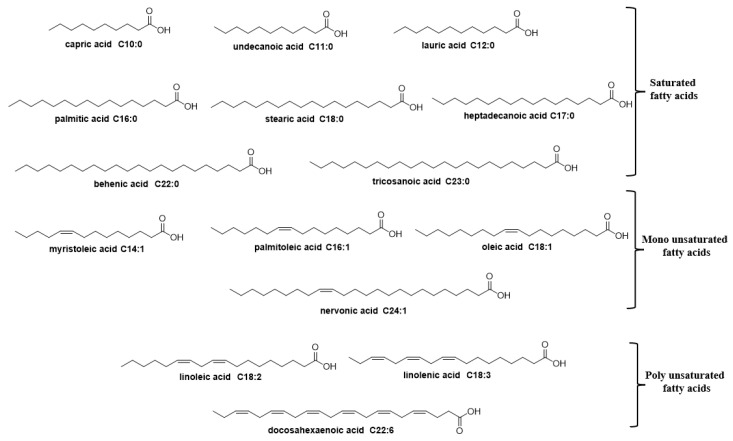
Structure of fatty acids used in this study.

In a first approach, we observed the presence of fatty acids such as palmitic acid, oleic acid, linoleic acid, and lauric acid, which made up over 50% of the fraction composition. To determine the efficiency of the fatty acids present in fraction 2 against both *B. cereus* and *C. acnes*, we decided to test commercial samples of the fatty acids palmitic, oleic, linoleic, and lauric acid as well as some of their available parent fatty acid analogues (Figure 1). The results are summarized in Table 3.

It clearly appears that fatty acids such as palmitic, oleic, and lauric acids do not present any activity against Gram-positive or Gram-negative bacteria. In contrast, linoleic acid demonstrated significant antibacterial effects, with MICs of 8 µg/mL against *S. epidermidis*, *S. aureus*, *B. cereus*, and *C. acnes* strains.

A subsequent study against numerous *C. acnes* strains, including an erythromycin-resistant one, was performed, demonstrating the general antimicrobial action of linoleic acid in respect of these bacterial strains (Table 4).

**Figure 2:**
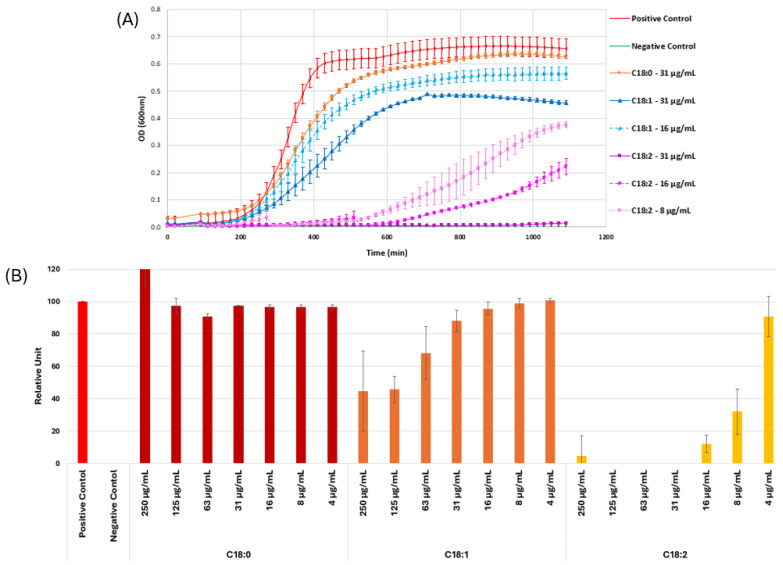
(**A**) Inhibition of growth of *B. cereus* ATCC11778 exhibited by C18:0, C18:1, C18:2 fatty acids used at concentrations ranging from 8 to 31 µg/mL. Bacteria were used as positive control and media as negative. (**B**) Growth rate index, determined by comparing the time it takes for a bacterial culture exposed to C18:0, C18:1, or C18:2 to enter the logarithmic growth phase relative to an identical bacterial culture that is not subjected to any stress.

The real-time growth inhibition profiles of C18:0, C18:1, and C18:2 fatty acids against *Bacillus cereus* ATCC 11778 were calculated to determine the importance of the structure of these different fatty acids on the antibacterial activity. At concentrations of 31 µg/mL for C18:0 and 16 µg/mL for C18:1, no or only weak inhibition of bacterial growth was observed. On the other hand, the growth of *B. cereus* was completely inhibited when linoleic acid (C18:2) was used at a concentration of 31 µg/mL. Significant growth inhibition was noted only with C18:2 at a lower concentration of 16 µg/mL, where inhibition occurred during the initial 10 h, but the bacteria eventually overcame the compound’s effects (Figure 2A). The behavior of the different fatty acids is more clearly illustrated in Figure 2B using the growth rate index, determined by comparing the time it takes for a bacterial culture exposed to C18:0, C18:1, or C18:2 to enter the logarithmic growth phase relative to an identical bacterial culture that is not subjected to any stress. C18:0 showed no effect on *B. cereus* growth, while C18:1 had a weak effect only at high concentrations. In contrast, C18:2 exhibited strong growth inhibition, even at low concentrations (8–16 µg/mL) (Figure 2B).

**Figure 3:**
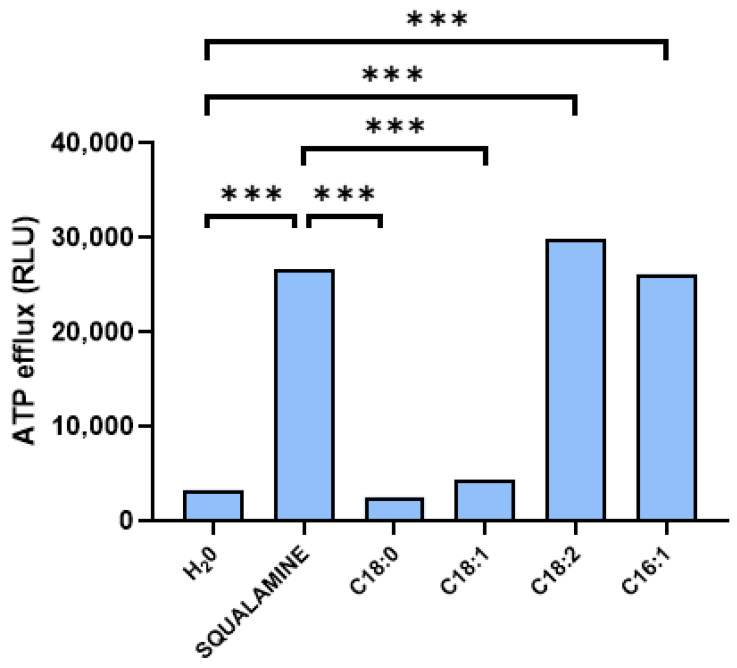
*B. cereus* membrane permeabilization based on rate of ATP efflux in the presence of water (negative control), squalamine (positive control), and C18:0, C18:1, C18: 2, and C16:1 fatty acids used at 100 µg/mL concentration. ANOVA followed by post hoc Tukey test were used for statistical analysis (*** *p*  ≤  0.001).

Finally, we measured extracellular ATP levels as an indicator of membrane permeability, where an increase in ATP outside the cell suggested membrane disruption (Figure 3). Squalamine was used as a positive control, and the kinetics of intracellular ATP release were monitored over 30 min to determine the maximum ATP efflux. In contrast, water, used as a negative control, along with fatty acids C18:0 and C18:1, showed no significant ATP release, indicating little to no effect on the bacterial membrane. However, fatty acids C18:2 and C16:1 triggered a rapid release of ATP, reaching 100% of the maximum efflux within 30 min, like squalamine.

## 4. Discussion

Propolis, a resinous substance crafted by honeybees, presented a variable composition principally based on plant source and climate conditions. The unsaturated fatty acid level found in propolis OFAP2 represents 51% of the total fatty acid composition, suggesting that this could constitute a beneficial source of unsaturated fatty acids in the diet. The fatty acid composition of propolis has been previously studied in countries such as Algeria [22], Turkey [23], Oman [24], and Cameroon [25]. However, no research has been conducted on the fatty acid composition of propolis samples collected in Rwanda and their potent antimicrobial activities. The identification of fatty acid compounds in our GC/MS analysis was definitively established by comparing the results to authentic standards of commercially available fatty acids. All of our data suggest that linoleic acid is the active component isolated from propolis OFAP2 against *C. acnes*. Of particular interest is the screening of commercially available fatty acids that we performed, where some, such as myristoleic, palmitoleic, docosahexaenoic, and linolenic acids, exhibited promising results against *C. acnes*, a relatively slow-growing, aerotolerant anaerobic Gram-positive bacterium associated with acne. Nevertheless, none of them presented a selectivity of action with respect to *C. acnes* and *S. epidermidis*, which can be problematic, since the latter is not merely a passive resident on skin, but actively primes the cutaneous immune response by preventing opportunistic pathogens to cause disease via colonization resistance. The mechanism of action of various fatty acids was then investigated by studying their membrane-permeabilizing and/or disrupting effects on the Gram-positive bacterium *B. cereus*, and not *C. acnes*. *C. acnes* is an anaerobic, Gram-positive bacillus whose growth requires an oxygen-free environment. However, the Tecan Spark Reader operates exclusively under aerobic conditions, making it unsuitable for direct testing against *C. acnes*. Consequently, our assay was performed with *B. cereus*, since the MIC values obtained against these bacteria matched those observed for *C. acnes*, validating its use as a comparable model in this context.

Our data suggested that C18:2 and C16:1 can disrupt the membrane integrity of *B. cereus* effectively. It is important to note, however, that while the concentration of these fatty acids in the study was high (100 µg/mL), their membrane-disrupting activity was observed at significantly lower concentrations, around 16 µg/mL suggesting an important interaction with the membrane of *B. cereus*. Thus, mono- and polyunsaturated fatty acids, due to their particular chemical structure, can interfere with bacterial cell membranes, preventing them from growing or multiplying, while saturated fatty acids do not appear to have this same effect. Fatty acids serve as the fundamental components of all lipid substances in the human body. Structurally, they are long-chain molecules that differ in the number of carbon atoms and the level of saturation in their chemical bonds. Essential fatty acids (EFAs) are particularly important, having long been recognized for their crucial role in human health and in the development of various diseases. Patients who are deficient in EFAs, especially those receiving intravenous fluids, often develop chronic scaly dermatosis characterized by increased skin water loss due to the compromised integrity of the skin barrier. However, essential fatty acids such as linoleic, linolenic, and arachidonic acids must be exclusively obtained through dietary sources [26]. In some cases, the absorption of topically applied linoleic acid has been sufficient to correct most clinical signs of essential fatty acid deficiency, while linolenic acid and arachidonic acid have not demonstrated the same effectiveness [27]. Acne is a persistent inflammatory condition, often causing disfigurement, that predominantly affects the face, neck, and upper torso in postpubertal men and women. Its development begins with the blockage of follicular structures due to changes in keratinization of the follicular epithelium. This environment promotes the proliferation of *C. acnes* within the follicles, leading to the breakdown of sebaceous triglycerides and wax esters into free fatty acids and glycerol. The resulting free fatty acids irritate the follicular epithelium, causing follicle rupture and initiating a series of inflammatory responses that manifest as acne papules, pustules, and cysts. Strauss et al. first observed that the excessive sebaceous secretion typical in acne patients leads to a localized deficiency of linoleic acid in the sebaceous glands [27]. These glands produce linoleic acid-depleted sebum, which they suggested could disrupt follicular keratinization and contribute to the formation of acne lesions. This hypothesis gained significant support from the work of Strauss et al., who analyzed linoleic acid levels in the sebum of patients treated with oral 13-cis retinoic acid [27]. This medication, known to markedly reduce both sebum production and sebaceous gland size, further validated the theory. Interestingly, the percentage of linoleic acid, which was low before therapy, increased dramatically during treatment, but returned to pretreatment levels once the drug was stopped and sebum secretion increased again. The clinical improvement in the disease seemed to correspond with the rise in sebum linoleic acid levels.

Finally, given the significant variability in the compounds found in different types of propolis, it is crucial to analyze each propolis sample individually in terms of its composition, rather than relying solely on general measures of biological activity, as is often done in the literature [28]. In fact, we recently studied a propolis sample collected just 20 km from the one examined here and found that it had a completely different GC-MS profile [20]. Notably, it contained an active compound (2,4-Di-*tert*-butylphenol) against *C. acnes*, which was absent in the propolis analyzed in this study. These findings highlight the importance of identifying the specific molecules responsible for biological activity, leading to a deeper understanding of the therapeutic potential of propolis.

## 5. Conclusions

In summary, we identified linoleic acid from Rwandan propolis OFAP2, which inhibited *Cutibacterium acnes* growth at a concentration of 16 μg/mL, even against the erythromycin-resistant strain, by disrupting the membrane integrity. Our data suggest that a cream formulation based on linoleic acid could be highly efficient for the treatment of acne. Studies in this direction are now underway, and results will be reported in due course.

## Figures and Tables

**Table 1:** Antimicrobial activities of propolis ethanolic extract OFAP2 from Rwanda.

	MIC (µg/mL)						
Sample	*E. coli* ATCC25922	*S. aureus* ATCC25923	*P. aeruginosa* PAO1	*B. cereus* ATCC11778	*C. acnes* DSM1897	*C. acnes* DSM30753	*C. acnes* DSM110516
OFAP 2	>256	>256	>256	16	16	16	32
F1	>256	>256	>256	256	256	256	256
F2	>256	>256	>256	16	16	16	16
F3	>256	>256	>256	256	256	256	256

**Table 2:** Molecules identified and their proportion in fraction 2 of propolis OFAP2.

Peak	Ret. Time (Min)	Area (A.U.)	Area (%)	Molecule
1	13.24	297,113	0.93	Undecan
2	14.61	446,913	1.41	Coumaran
3	14.72	160,440	0.50	ND
4	15.58	441,560	1.39	Pyrocatechol or resorcinol
5	15.68	471,737	1.48	1-Dodecene
6	17.18	1,547,578	4.87	dl-Mevalonic acid lactone
7	18.96	328,518	1.03	*p*-Formylphenol
8	21.20	1,842,071	5.79	*p*-Salicylic acid
9	22.08	753,073	2.37	Lauric acid
10	22.63	825,163	2.59	Caryophyllene oxide
11	23.30	750,742	2.36	Benzoic acid
12	23.57	622,666	1.96	Alpha-cadinol
13	23.42	5,451,717	17.14	Palmitic acid
14	33.87	686,184	12.16	Linoleic acid
15	34.16	5,750,531	18.08	Oleic acid
16	34.92	1,809,658	5.69	ND
17	38.83	3,956,525	12.44	Thunbergol A
18	39.66	3,236,609	10.18	Thunbergol B
19	43.20	2,424,825	7.62	Ferruginol

**Table 3:** Antimicrobial activities of fatty acids against different bacterial strains.

	MIC (µg/mL)														
Cpd	C10:0	C11:0	C12:0	C16:0	C17:0	C18:0	C22:0	C23:0	C14:1	C16:1	C18:1	C24:1	C18:2	C18:3	C22:6
Strains															
*S. aureus* ATCC25923	>64	>64	>64	>64	>64	>64	>64	>64	64	16	>64	>64	8	16	16
*S. epidermidis* CIP81.55	>64	>64	>64	>64	>64	>64	>64	>64	16	8	>64	>64	8	8	16
*B. cereus* ATCC11778	>64	>64	>64	>64	>64	>64	>64	>64	8	8	>64	>64	8	8	16
*E. coli* ATCC25922	>64	>64	>64	>64	>64	>64	>64	>64	>64	>64	>64	>64	>64	>64	>64

**Table 4:** Antimicrobial activities of linoleic acid against numerous *C. acnes* bacterial strains.

Sample	CMI (µg/mL)					
	*C. acnes* DSM1897	*C. acnes* DSM110512	*C. acnes* 110516 (ERY-R)	*C. acnes* DSM110517	*C. acnes* DSM110528	*C. acnes* DSMA179
Linoleic acid	16	16	16	16	16	16

## Data Availability

Dataset available on request from the authors.

## Supplementary Materials

The following supporting information can be downloaded at: https://www.mdpi.com/article/10.3390/cimb47030162/s1.
