# Supplementary material for: Linoleic Fatty Acid from Rwandan Propolis: A Potential Antimicrobial Agent Against Cutibacterium acnes
Source: Curr Issues Mol Biol. 2025 Feb 27;47(3):162. doi: 10.3390/cimb47030162 (PMC11941583; doi:10.3390/cimb47030162)
Supplement: Supplementary file 1 [file cimb-47-00162-s001.zip › cimb-3469846-supplementary.pdf]

Supplementary materials

Linoleic fatty acid from rwandan propolis: a potential antimicrobial agent against *Cutibacterium acnes*

[1] Aix Marseille Univ, INSERM, SSA, MCT, 13385 Marseille, France. E-mail : **jean-michel.brunel@inserm.fr**
[2] Observatoire Français d 'Apidologie (OFA), La Garniere, Route de Mazaugues, 83136 Mazaugues, France
[3] C2VN, Aix Marseille Univ, INSERM 1263, INRAE 1260, 13385 Marseille, France.

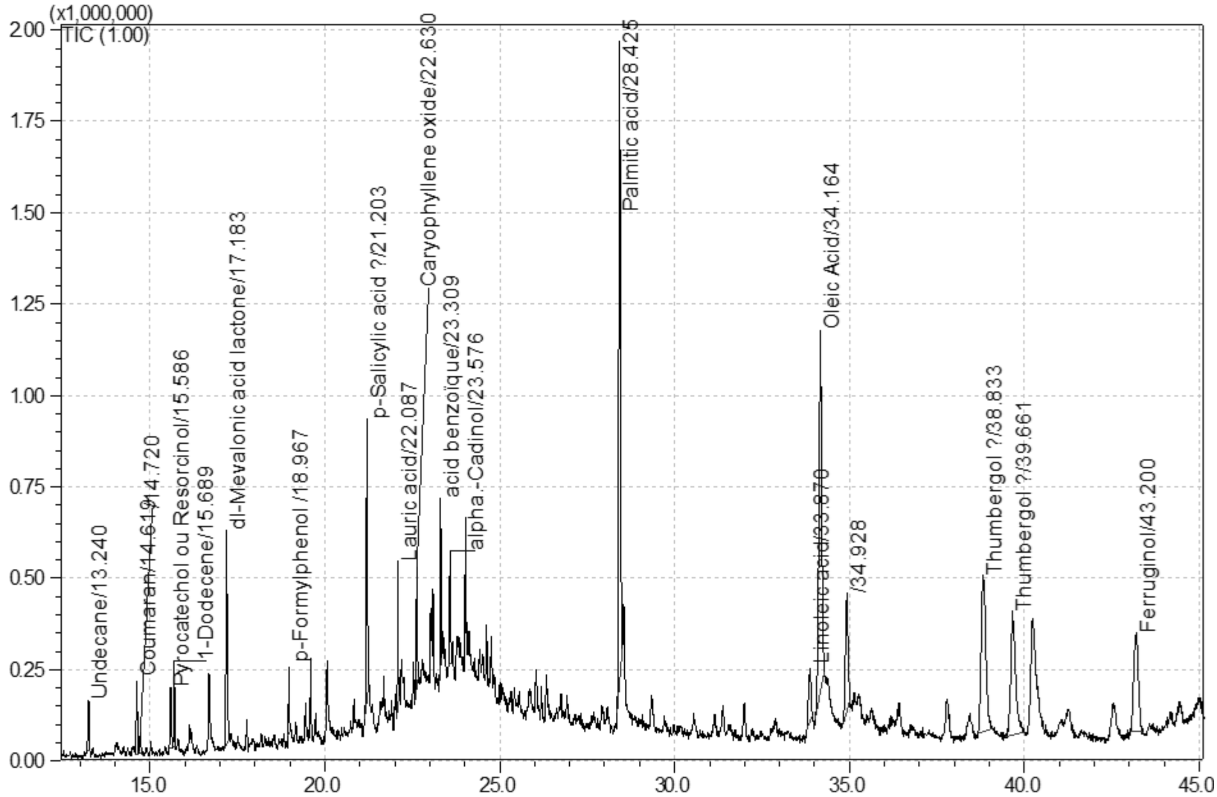

Figure S1. GC chemical profiling of fraction 2 of propolis OFAP2

MIC (µg/mL)			
	C. acnes DSM1897	*C. acnes* DSM30753	*C. acnes* DSM110516
Doxycycline (in water)	0.5	0.25	0.5
Doxycycline in ethanol(70%)	0.5	0.25	0.5
Erythromycine (in water)	0.25	0.125	0.25
Erythromycine in ethanol (70%)	0.25	0.125	0.25

Table S1. MIC of doxycycline and eryhtromycine against 3 *C. acnes* strains solubilized in water or ethanol (70%).
